# High-resolution behavioral mapping of electric fishes in Amazonian habitats

**DOI:** 10.1038/s41598-018-24035-5

**Published:** 2018-04-11

**Authors:** Manu S. Madhav, Ravikrishnan P. Jayakumar, Alican Demir, Sarah A. Stamper, Eric S. Fortune, Noah J. Cowan

**Affiliations:** 1 Mind/Brain Institute, Johns Hopkins University, Baltimore, Maryland USA; 2 Mechanical Engineering Department, Johns Hopkins University, Baltimore, Maryland USA; 3 Federated Department of Biological Sciences, New Jersey Institute of Technology, Newark, New Jersey USA

## Abstract

The study of animal behavior has been revolutionized by sophisticated methodologies that identify and track individuals in video recordings. Video recording of behavior, however, is challenging for many species and habitats including fishes that live in turbid water. Here we present a methodology for identifying and localizing weakly electric fishes on the centimeter scale with subsecond temporal resolution based solely on the electric signals generated by each individual. These signals are recorded with a grid of electrodes and analyzed using a two-part algorithm that identifies the signals from each individual fish and then estimates the position and orientation of each fish using Bayesian inference. Interestingly, because this system involves eavesdropping on electrocommunication signals, it permits monitoring of complex social and physical interactions in the wild. This approach has potential for large-scale non-invasive monitoring of aquatic habitats in the Amazon basin and other tropical freshwater systems.

## Introduction

The study of animal behavior often requires identification and localization of individuals as they move through the environment. In the laboratory and in certain field conditions, this information can be extracted from video recordings of individual organisms during complex interactions^1,2^. Numerous methodologies have been developed that enable automated and semi-automated video tracking of individuals over a large range of spatial scales, ranging on the order of millimeters (e.g. *C. elegans*), to tens of meters (e.g. bats)^3–12^. Tracking over larger scales, e.g. kilometers, has been accomplished using devices attached to organisms^13–15^. There are, however, many species and environments in which these tracking techniques are infeasible, including certain aquatic environments and dense forests, as well as for species in which attaching tracking devices to each individual is not possible.

Many species that are difficult to track using video, however, may betray their locations via the production of signals, such as sound or electricity, that can be localized using computational techniques. Audio systems that monitor autogenous acoustic signals, such as whale songs or bat calls, using grids of microphones have been used to track some terrestrial and aquatic species^16–20^.

We developed a sensor array and analytic tools for measuring the positions and electrical behaviors of weakly electric fishes. These fish species are widespread throughout the Amazon basin and in certain river systems in Africa. Measuring and monitoring the numbers of individuals, spatial movements and distributions, and social interactions of these fish will provide insights that can be used in the context of ecology and conservation of sensitive Amazonian habitats. These measurements are also critical for interpreting data from neurophysiological studies of electrosensory control circuits in this important neuroethological model system^21–24^.

Weakly electric fish use a specialized electric organ to continuously produce electric fields that are detectable at distances of up to 2 m^21,25^. For many species (so-called wave-type fishes) this electric organ discharge (EOD) is pseudo-sinusoidal with fundamental frequencies that range from below 50 Hz to above 1500 Hz. These nocturnal fish commonly live in turbid water and in complex root and littoral habitats, where video tracking is generally not possible. We have developed a recording system and computational approach to track multiple wave-type electric fish that relies solely on their EOD signals. Using this system, we tracked *Eigenmannia virescens*, a species of Gymnotiform fish, in both laboratory and field settings. Our method is designed to make long-term behavioral recordings of these animals in the wild.

We capture the electric signals using a grid of amplified electrodes that are deployed in the fish’s habitat. Our analytic approach is composed of two steps. In the first step, the algorithm identifies a set of signal features (e.g. frequency, harmonic amplitude ratio), that are unique to each individual fish. In the second step, the algorithm estimates the location of each fish by solving an inverse problem based on the sensor geometry and an electrostatic dipole model. In theory, each fish’s location could be estimated analytically by inverting the signal propagation. However, even if the fish were a perfect dipole in an infinite, placid lake, the transformation from fish position to the array of sensor measurements is highly nonlinear, rendering the inverse problem challenging. Moreover, unpredictable sources of physical and biological variability (e.g. turbulent clouds of silt, fish and other objects through the grid, body bending) dynamically alter electric fields that add “noise”. As a result, we believe the inverse problem is best addressed using statistical estimation techniques.

Our statistical approach is to estimate each fish’s position and orientation using a particle filter.  In essence, the particle filter simulates thousands of signal sources (particles) in the environment, and compares the simulated readings at the sensors from these sources to the actual measured values. The particles with the closest readings to the actual measurements are assigned higher “weight”, and the location estimate is the weighted mean of particle locations.

## Results

**Figure 1:**
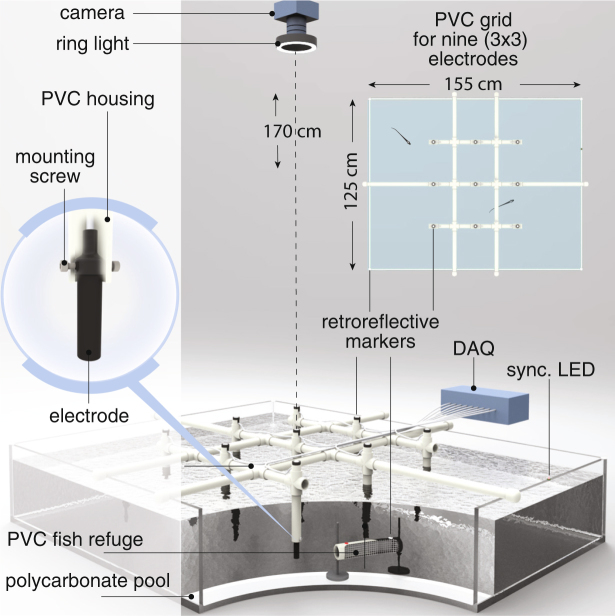
The laboratory grid setup. A 3 × 3 grid of electrodes (50 cm inter-electrode spacing) are mounted to a PVC support structure, which was in turn mounted to the edges of an acrylic tank. In the TUBE condition, fish are enclosed in tubular refuges and placed near the bottom of the tank. In the FREE condition, fish can swim freely throughout the tank. A ring light around the camera mounted above the tank illuminates retroreflective markers on the electrodes. The electrical signals are captured and subsequently recorded by the DAQ.

Our approach relies on recordings made with an electrode grid system (Fig. 1) and a two-step algorithm to extract the identities and positions of individuals in a laboratory tank in which the positions of the fish were also tracked via video recordings. We subsequently validated the system at a field site in Brazil, in which fish were restrained in mesh tubes at known locations within the grid.

The tracking system involves three steps: (1) capturing the data using an array of electrodes placed in the habitat of the animals, (2) extracting the parameters of the electric signals (e.g. fundamental frequencies and harmonics) of each individual fish, and (3) tracking the spatial position and orientation (pose) of each fish with respect to the grid geometry.

### Validation of frequency tracking

The frequency tracking algorithm is semi-automated. The computer extracts potential frequency tracks produced by individual fish by identifying peaks in amplitudes of Fourier transforms of the electrode data. These frequency tracks are then superimposed on the spectrogram of the recorded data. The user then selects those tracks that match the frequency bands in the spectrogram - each band corresponds to the EOD of an individual fish.

Frequency tracking of signals recorded in the laboratory required little human intervention, because of the high signal-to-noise ratio, and due to the reduced electrosocial behaviors that *Eigenmannia* and other species exhibit in large laboratory tanks. *Eigenmannia* maintained almost constant EOD frequencies that were typically separated by more than 5 Hz from other fish in the tank.

In contrast, we observed more complex electrosocial behaviors and increased interference, including 60-cycle noise, at our field sites. Fish routinely changed their frequencies over a range of more than 20 Hz, and routinely crossed or shared EOD frequencies for periods of up to tens of seconds. The algorithm is robust to such crossings, and the interface allows the user to reassign frequency tracks as needed. For fish that shared the same frequency for tens of seconds, we were able to use both small independent deviations in EOD frequencies of each fish and differences in amplitude across the electrode array to identify individuals.

As this algorithm involves human validation, no further tests of the performance of the frequency tracking were conducted. We extensively and explicitly validate the spatial tracking which implicitly validates the frequency tracking.

### Validation of spatial tracking

The second component of the algorithm, spatial tracking, is fully automated. We validated the pose of each fish estimated by the tracking algorithm by comparing against the pose measured through image processing of overhead video. We performed two types of experiments in the acrylic laboratory tank. In TUBE trials, one or three fish were enclosed in PVC tubes which were stationary during the course of one trial. In FREE trials, one or three fish swam freely in the tank, and we chose epochs where one or more fish were swimming through the grid. A detailed description of the experimental setup and data collection is provided in the methods section.

### Laboratory trials

When the output of the spatial tracking algorithm, i.e. the electrode-tracked pose was compared to the camera-tracked pose, we observed that the estimate of fish pose deteriorated (i.e. the error increased) when the fish were close to the tank boundary. We attribute this to the insulating acrylic walls of the tank, which distort the signals from any sources close to it. Because of these boundary effects, we partitioned the data into two sections based on the actual position of the fish: within the grid and outside the grid. Fish are considered to be within the grid if their video-tracked position is within the square defined by the outermost electrodes.

**Figure 2:**
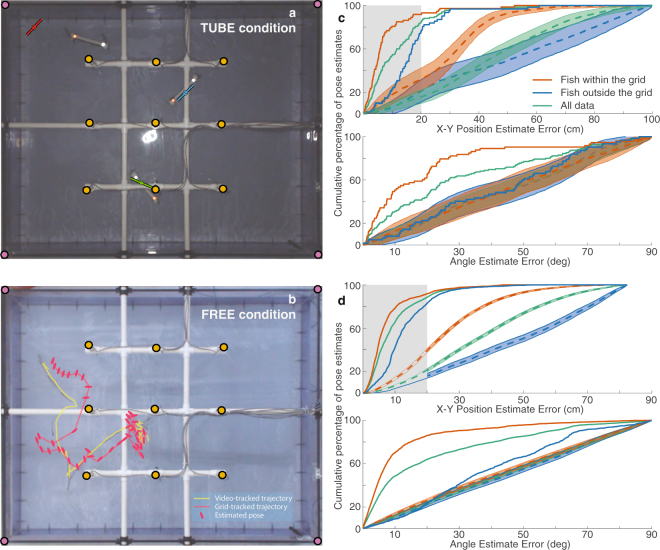
Results of spatial tracking. (**a**) Overhead view of one three-fish TUBE trial. The mean position from spatial tracking is shown using the circle markers with orientation indicated by the lines. The radius of the circle corresponds to two standard deviations of the X-Y position estimate. (**b**) Overhead view of one single-fish FREE trial, constructed by superimposing multiple keyframes from the video. Trajectory of the fish tracked from the video along with the trajectory and orientations of the fish estimated by the electric tracking system. (**c**) Cumulative error plots (solid curves) of position (top) and angle (bottom) for all TUBE trials. For position plots, the length of the tube (20 cm, ≈1.5 fish body lengths) is shaded in grey. Errors are divided into instances when the fish was within the grid, outside the grid, and all data taken together. The dashed curves show the mean of the shuffled cumulative error distributions and the shaded areas indicate the the 0.001 and 0.999 quantiles of the shuffled data, equivalent to 99.8% CI (see Statistical Methods). (**d**) The same curves for FREE trials.

In the TUBE condition, for fish within the grid, a majority of positions and orientations estimated by our method fall remarkably close to the actual pose of the fish. Specifically, within the grid, more than 90% of the tracked positions were within 20 cm (≈length of the tube, ≈1.5 fish body lengths) of the video-tracked positions. More than 80% of the tracked orientations were within 30° of the video-tracked orientations (Fig. 2(c)). Data within the grid have better (lower) cumulative error curves than data outside the grid, and the combined data have a cumulative error curve in between the two.

In the FREE condition, the tracking error is nearly identical to that seen for the restrained fish; more than 90% of the position errors were within 20 cm and more than 90% of the tracked orientations were within 30° of the video-tracked orientations (Fig. 2(d)). The cumulative error curves also have the same performance relationships.

**Figure 3:**
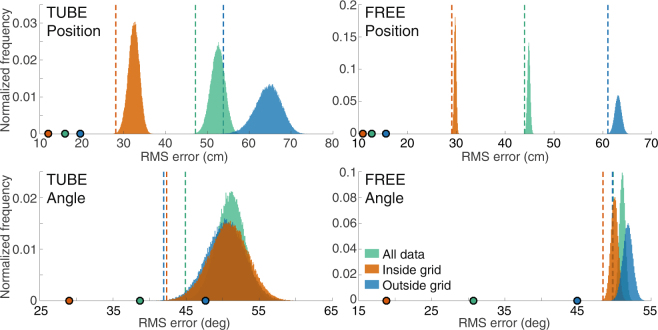
Shuffled error test of spatial tracking. Position (top) and Orientation (bottom) errors are shown for TUBE (left) and FREE (right). The normalized histograms of the shuffled RMS error distributions (see Statistical Methods) of all data are shown, as well as the subsets of data within and outside the grid. The dotted vertical lines indicate the 0.001 quantile lines (equivalent to 99.9% CI) of the distributions of the corresponding colors. The points on the x-axis represent the RMS errors of the true (unshuffled) data of the corresponding colors.

We compared the tracking errors to two different test statistics on a dataset where the video-tracked poses were randomly shuffled against the electrode-tracked poses (see Statistical methods for details). The shuffled cumulative error distributions for data within the grid, data outside the grid, and all data, along with their 0.1% and 99.9% quantiles are plotted in Fig. 2(c) and (d). The shuffled distributions appear to have the same relative relationships as the true error curves. The cumulative error curves of the true data are lower than the shuffled distributions in all cases except the orientation error for data outside the grid. This indicates that our algorithm cannot determine orientation of the fish accurately when the fish are restrained outside the grid boundaries. This is in fact confirmed and quantified by the second statistic, the root-mean-square (RMS) error between the set of video-tracked and electrode-tracked position and orientation estimates. Figure 3 plots the shuffled RMS error distributions for positions and orientations in the TUBE and FREE conditions, along with their 0.1% quantile, and the RMS errors of the true data. In all cases except TUBE orientation outside the grid, the true data have significantly lower RMS errors than the distributions. For these cases, we can reject the null hypothesis that the tracking error of the actual data was statistically no different from a random permutation of the locations of the fish. However, in most cases, especially when the fish are freely swimming and within the grid, our algorithm performs orders of magnitude better than chance.

### Field trials

**Figure 4:**
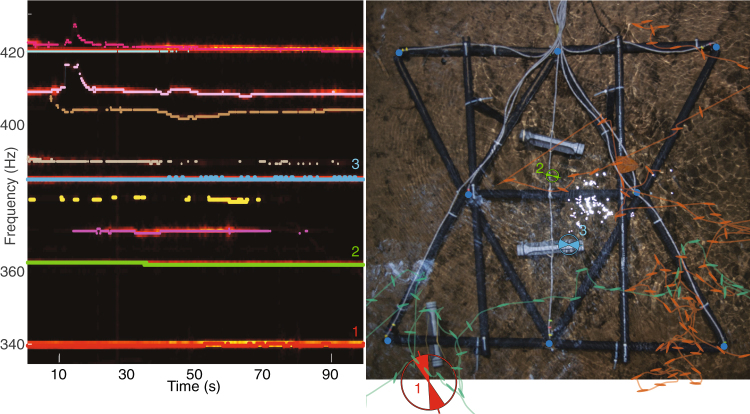
A grid of 8 electrodes was deployed at a field site in Brazil (right). Three fish were captured, their baseline EOD frequencies recorded, and placed in tubes. Signals were recorded from the grid electrodes, while other free electric fish swam within the grid. The spectrogram of the recorded data (left) reveals several tracks, including those belonging to the restrained fish (marked as 1–3). We traced the locations of all the *Eigenmannia* that entered the grid area during the trial. The trajectories of two freely swimming fish are shown as examples on the right, with their orientations indicated at several locations. For the restrained fish, the mean and two standard deviations of the position estimate is marked using circles, and the mean and two standard deviations of the orientation estimate is marked using wedges within the circles.

An 8-electrode grid with a 50 cm.inter-electrode spacing was deployed in the Lapa river within 200 m of the entrance of the Terra Ronca I cave (13°44′06.8″S 46°21′29.3″W, Goiás, Brazil). A characteristic of this field site was crystal-clear water, which allowed us to video the grid from above and under the water. The depth of the water under the grid ranged from 5 cm to 40 cm. The conductivity of the water has been reported to range between 15 and 34 uS/cm^26^. We performed four 100s recordings from the field site (Fig. 4). In each recording, three fish with known EOD frequencies were restrained in tubes at known locations and orientations within the grid. However, other *Eigenmannia* freely swam in and around the grid. Interestingly, the fish in tubes maintained nearly constant EOD frequencies, whereas the free fish produced complex excursions in EOD frequency (Fig. 4).

We spatially tracked fish in the wild using the same parameters as those used for tracking fish in the lab. A site photo with overlaid spatial estimates is shown in Fig. 4. The spatial estimates varied over the 100 s long trials and we characterized the distribution of the estimates across time. The distribution of each fish’s estimate for each data set was non-normal (Royston’s Multivariate Normality Test, 5% significance level). To quantify the error in the estimate over time, we computed the root-mean-squared-error (RMSE) for each fish in each dataset (see Table 1). Errors were typically within 1.5 body lengths (10/12 estimates) and 15° (8/12 estimates), and never worse than 3 body lengths. As with the tracking in the lab, the positions and orientations estimated by our method fall close to the actual pose of the fish.

**Table 1:** Root-mean-squared-errors (RMSE) of position and orientation estimates in the field.

Dataset		Position	Orientation
		RMSE (cm)	RMSE (deg)
TerraRonca_01	Fish 1	6.6	9.9
	Fish 2	10.6	3.1
	Fish 3	16.5	12.6
TerraRonca_02	Fish 1	**33.8**	**57.9**
	Fish 2	**43.9**	**50.1**
	Fish 3	15.6	6.1
TerraRonca_03	Fish 1	13.1	**22.9**
	Fish 2	8.6	5.6
	Fish 3	7.6	4.5
TerraRonca_04	Fish 1	13.1	6.6
	Fish 2	19.3	**28.5**
	Fish 3	17.3	4.7

We performed four 100 s recordings in the field (TerraRonca_01 through TerraRonca_04), each of which had three fish restrained in tubes, and estimated their poses. The position RMSE which are not within the length of the tube (20 cm, ≈1.5 fish body lengths) and angular RMSE which are not within 15° are shown in bold.

## Discussion

Weakly electric fish are nocturnal and commonly live in silty, complex habitats, making video tracking generally impossible. Fortunately, these fish betray their position by continuously generating autogenous electric signals for communication and for detecting and characterizing nearby objects. We eavesdropped on these signals using a grid of custom electrodes as the fish swam in their natural Amazonian habitats. We tracked each individual’s electric field frequency and used the relative distribution of amplitudes and phases across the electrodes to estimate the 3D position and orientation of each fish. In a sense, we tackled a similar inverse problem as the electrosensory system of the fish–finding the location and orientation of objects in the environment using spatially distributed measurements of electric fields, an idea that has also been explored in bio-robotic electric navigation^27,28^.

Previous work demonstrated the feasibility of using a multi-electrode array to spatially localize pulse-type weakly electric fishes^29^. This approach, designed for behavioral observation in a laboratory setting, is similar to ours in that simulated electrode readings based on a dipole model are compared with actual readings from the fish. They take advantage of the known geometry of the tank and water surface boundaries to account for boundary effects in the simulated readings. A shallow water depth also allowed them to use a planar dipole model.

Field recordings, by contrast, occur under much more uncontrolled conditions, such as environmental noise sources corrupting the signal, unknown boundary conditions and variable water depth. Since the electrode grid is constructed on site under less than ideal conditions, there can also be small uncertainties about the geometry of the grid of electrodes. We use a Bayesian approach, specifically the particle filter, to deal with such noisy recordings. This allows us to construct the posterior probability density function of the fish’s state based on all available information. This distribution would, in principle, yield an optimal estimate of the state as well as a measure of confidence^30^.

The spatiotemporal resolution and accuracy of the grid system permits the observation of currently unknown parameters of locomotor-related behaviors, particularly social behavior. The system can resolve the numbers and movements of fish in groups, which is known to vary between species^25,31,32^. We will, for the first time, be able to identify the transit of individuals across territories and through groups, providing insight into the parameters that govern these differences in behavior (see^33^). However, close interactions (less than one body length distance), which can include body contacts, biting, and complex poses^34^, cannot be determined using this system. These important social behaviors are of interest to biologists but are unlikely to be resolvable using the current technology.

Weakly electric fish are distributed through much of the Amazon basin and are known to be sensitive to environmental perturbations^35,36^ making them a bellwether for changes to critical Amazonian habitats. The technology presented in this paper, which takes advantage of the continuous electrical signals produced by these animals, can be scaled for widespread monitoring of these fish and therefore of environmental impacts of human activity: the custom amplifiers are composed of a simple, low-power, inexpensive circuits, readily integrated into autonomous systems that automatically capture and upload data. These data can be analyzed to assess behavioral activity, species distributions and diversity.

## Methods

Adult *Eigenmannia virescens* (10–15 cm in length, EODs between 346–452 Hz) were obtained from commercial vendors. The fish were housed in group aquarium tanks that had a water temperature of approximately 27 °C and a conductivity in the range of 150–500 *μ*S/cm^37^. All experimental procedures were approved by the Johns Hopkins Animal Care and Use Committee and the Rutgers Institutional Animal Care and Use Committee, and followed guidelines established by the National Research Council and the Society for Neuroscience. Permits to conduct this research in Brazil were granted to Dr. Maria Elina Bichuette.

### Electrode design

**Figure 5:**
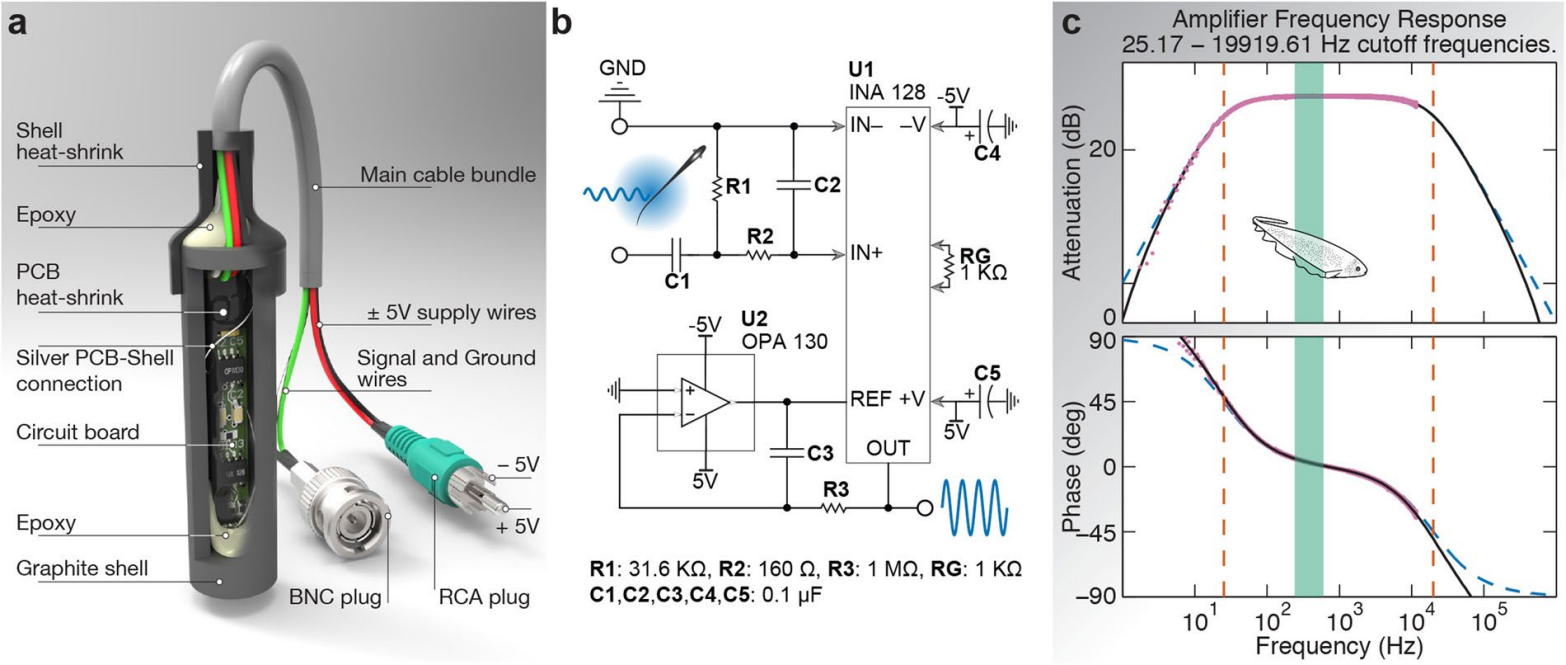
(**a**) Mechanical design of the electrode (**b**) Schematic of the individual amplifier with band-pass input. (**c**) Bode plot of the whole amplifier. The dashed blue lines represent the theoretical response given an ideal op-amp. The solid black line represents the theoretical response incorporating the manufacturer’s models for both the instrumentation amplifier (INA128) and the reference buffer (OPA130). The magenta dots represent experimental data.

We designed and built custom amplifiers to record the specific electric signals produced by *Eigenmannia*. These fish produce an EOD in the frequency range 200 to 750 Hz with measured amplitude on the order of mV. Correspondingly, the amplifier circuitry (Fig. 5a) consists of a passive band-pass filter (≈25–20000 Hz) at the input, an instrumentation amplifier with a gain of ≈50, and an op-amp buffer at the reference which acts as a high-pass filter (≈1.6 Hz) to mitigate the AC-coupling.

The input band-pass filter reduces low- and high-frequency noise signals that contaminate the fish’s electric field signal. The filter cutoff frequencies (≈25–20000 Hz) are chosen to be well outside of the *Eigenmannia Virescens* frequency range so that relevant data are not affected.

The mechanical design of an electrode assembly is presented in Fig. 5a. We wrapped the amplifier circuitry in a heat- shrink tube and applied epoxy at both ends after placing it into a cylindrical graphite shell in order to waterproof it. Once submerged in the water, the graphite shell acts as the electrode and conducts the signal to the amplifier board via a 27-gauge silver wire. Power for the amplifier, ground, and the output signals are carried in a four-channel cable bundle whose length depends on the location of the electrode in the grid. Finally, the open cap of the graphite shell and the main cable interconnection is sealed with an outer heat-shrink tube. Standard BNC and RCA plugs are used to connect the amplifier to the data acquisition device and power supply respectively.

We tested our electrodes by recording their responses to a range of frequencies 0.5 Hz–15 KHz at 0.1 V. This was accomplished by generating frequency sweeps (chirps) from a function generator. We sampled both the input and output responses at 25 KHz using a data acquisition device. The theoretical cutoff frequencies of our band-pass filter are 25.2 Hz and 19.9 KHz. The theoretical frequency response curve is plotted in Fig. 5c, along with the matching response from the chirp test. The *Eigenmannia Virescens* frequency range is depicted in the green shaded area where the attenuation is 0.50 (−6 dB) and phase is 2.4°.

Once electrodes were constructed, we assembled them into an array which could be easily deployed at field sites. Our field recording sites were shoreline edges, grass beds, and tree root systems that were reached by canoe. The grid array facilitated rapid assembly, transport, and deployment at these types of sites.

Electrodes were attached to a 1.5 m PVC grid with electrode spacing of 50 cm Each electrode was held ~10 cm below the surface using a perpendicular PVC tube. The entire grid was suspended along the water surface using floatation fixtures in the corners and along the PVC piping. The electrode-amplifiers were powered by a common ±1.5 V power supply and grounded via a carbon rod at least 1 m away from both the grid and from the water’s edge.

Data were recorded with a Micro1401 DAQ with 12-channel expansion using Spike2 software (CED, Cambridge, U.K.).

For laboratory experiments a smaller 9 electrode grid (with the same electrode spacing) was constructed due to space constraints of the tank. All data recording methodology was identical.

### Oscillating dipole model

To analyze the data obtained with the electrode grid, we need a model of the electric field generated by the fish. For our algorithms, we modeled *Eigenmannia* approximately as an oscillating current dipole. This is consistent with models constructed from spatial measurements of the electric field of *Eigenmannia*^38,39^. We made the following assumptions in approximating the fish to be a dipole:

1. *The dipole length is small relative to the grid spacing*. If a dipole is sufficiently close to an electrode such that *r*≫*d* does not hold true, we can safely assume that for a grid of electrodes with spacing larger than *d*, the dipole is sufficiently far away from almost all other electrodes such that *r*≫*d* holds true for them. We observed that with the number and spacing of electrodes present in our grid, a distortion on any one electrode has an insignificant influence on the spatial tracking error.
2. *The dipole is horizontal*. We observed that *Eigenmannia* in laboratory tanks almost always oriented themselves horizontally, i.e. their anterior-posterior axis is held parallel to the water surface. We have also seen this anecdotally at our field sites. Thus, we specify the fish’s spatial location using four coordinates: the spatial position and the orientation in the *x* − *y* plane.

Consider an ideal current dipole, a source-sink pair of equal, but time- varying strength *I*(*t*), separated by a small distance *d* at the origin, oriented along the *x* axis. Using the assumption that *r*≫*d*, the potential due to this dipole at a point with polar co-ordinates (*r*, *θ*) in the plane defined by the dipole line and the point can be approximated by

1 $${\rm{\Phi }}=\frac{I(t\mathrm{)\ }d}{4\pi \sigma }\frac{cos\theta }{{r}^{2}}=KI(t)\frac{cos\theta }{{r}^{2}}$$

where *σ* is the conductivity of the medium. In *Eigenmannia*, we observed that contributions to the electric field oscillation can be well approximated by the first two harmonics, i.e.

2 $$I(t)=A\,\cos \,\mathrm{(2}\pi ft+\psi )+{\rm{\gamma }}A\,\cos \,\mathrm{(4}\pi ft+\xi )$$

where *f* is the fundamental frequency, *A* is the amplitude of the fundamental harmonic, *γ* is the ratio of the amplitude of the second harmonic to the fundamental, *ψ* is the phase is of the fundamental, and *ξ* is the phase of the second harmonic. Equations (1) and (2) make the quasi-static assumption that, for a given time window, the location (*r*, *θ*) and frequency *f* of each dipole source is stationary.

Each fish, $j=\mathrm{1,2,}\ldots {n}_{{\rm{fish}}}$, induces a potential ${{\rm{\Phi }}}_{i}^{j}$ at each electrode, $i=\mathrm{1,\; 2,}\ldots {n}_{{\rm{elec}}}$. This potential can be calculated by replacing (*r*, *θ*) with the pairwise configuration of the *j*^th^ fish to the *i*^th^ electrode $({r}_{i}^{j},{\theta }_{i}^{j})$, as well as substituting fish-specific current waveform parameters $({A}^{j},{\gamma }^{j},{\psi }^{j},{\xi }^{j},{f}^{j})$. Thus, for a given time window, we have:

3 $$\begin{array}{rcl}{{\rm{\Phi }}}_{i}^{j}(t) & = & {K}^{j}\frac{\cos \,{\theta }_{i}^{j}(t)}{{r}_{i}^{j}{(t)}^{2}}[{A}^{j}\,\cos \,(2\pi {f}^{j}t+{\psi }^{j})+{\gamma }^{j}{A}^{j}\,\cos \,(4\pi {f}^{j}t+{\xi }^{j})]\\  & = & {a}_{i}^{j}\,\cos (2\pi {f}^{j}t+{\psi }^{j})+{\gamma }^{j}{a}_{i}^{j}\,\cos (4\pi {f}^{j}t+{\xi }^{j})\end{array}$$

The amplitude ratio *γ*^*j*^ is invariant for each dipole. The spatial location of dipole *j* with respect to electrode *i* is contained in the term ${a}_{i}^{j}$. The potentials from all the dipoles sum to form the total potential at electrode *i*:

4 $${{\rm{\Phi }}}_{i}(t)=\sum _{j\mathrm{=1}}^{{n}_{{\rm{fish}}}}{{\rm{\Phi }}}_{i}^{j}(t\mathrm{).}$$

If the different dipoles are oscillating at different frequencies *f*^*j*^, these frequencies as well as their amplitudes at each electrode ${a}_{i}^{j}$ can be estimated using Fourier analysis of the measured signals Φ_*i*_. These amplitudes can then be analyzed over all the electrodes *i* to estimate spatial position at time *t*. By sliding the quasi-static time window, this analysis can be extended to the entire period of measurement.

Here we present a two-step algorithm to estimate the time-varying absolute position in three dimensions, $(x{(t)}^{j},y{(t)}^{j},z{(t)}^{j})$, planar orientation, ${\theta }^{j}(t)$, and oscillation frequency, $f{(t)}^{j}$ of each of the dipole sources indexed by *j*, given the set of potential measurements, ${\{{{\rm{\Phi }}}_{i}(t)\}}_{i\mathrm{=1}}^{{n}_{{\rm{elec}}}}$. Note that the electrodes are deployed at known locations on a rigid (PVC) grid. For example, see Fig. 1.

### Step 1: Frequency localization of multiple oscillating signals

**Figure 6:**
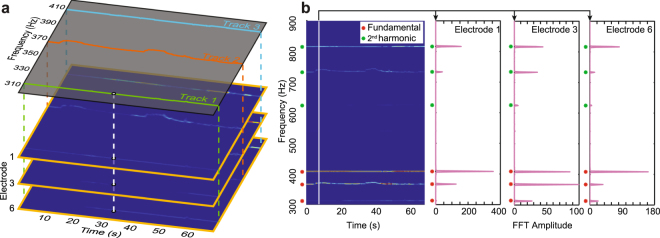
Representation of spectrogram data and extracted frequency tracks. (**a**) In the plots for each channel (electrode), the color denotes the amplitude of the signal at each frequency and at each time. This data over all channels is used to extract frequency “tracks”, which are continuous traces of frequencies identified over time. Three tracks are extracted from this dataset. (**b**) Spectrogram amplitudes and harmonics. At each time instant (solid white line), the amplitudes from three representative electrodes are shown. In the presence of multiple frequency components with harmonics, these electrode amplitudes will typically show a peak at the fundamental frequency, and another (often smaller) peak at the second harmonic. In this data, fundamental peaks (red asterisks) and second harmonic peaks (green asterisks) from three fish are indicated in all three electrode amplitude traces. Higher harmonic peaks also exist, but are typically weaker and are not used in the analysis and therefore not shown.

This step identifies the time-varying EOD signal parameters of each individual fish given measurements from a shoal of unknown size. The resultant “frequency tracks” represent the electric output of each individual (Fig. 6); together these tracks constitute the electrosocial milieu each animal experiences.

#### Step 1(a): Detect harmonic signatures

For electrode *i* and for a window centered around time *t*, we compute the short-time Fourier transform (STFT) of ${{\rm{\Phi }}}_{i}(t)$ using the spectrogram() function in Matlab. The result (Fig. 6) is a three-dimensional array of complex numbers, indexed across frequency, time, and electrode. At time *t* and for electrode *i*, we define a harmonic “signature” as a peak of the Fourier magnitude, which falls within a specified frequency range and above a specified threshold, *τ*_1_. Each signature should also have peaks at the second harmonic above a threshold, *τ*_2_. The ratio ${\tau }_{2}:{\tau }_{1}$ is chosen heuristically based on typical amplitude ratios, *γ*^*j*^, we have seen in laboratory experiments; see Equation (3). For finding signals corresponding to the weakly electric fish *Eigenmannia*, we assume the fundamental frequency lies between 200–700 Hz and set the amplitude threshold ratio to ${\tau }_{2}/{\tau }_{1}=\mathrm{1/8}$. The threshold *τ*_1_ was selected manually depending on the noise level of the data, and the strength of the fish EOD signal at the electrodes. This value also corresponds to the minimum of previous measurements of harmonic amplitude ratios in *Eigenmannia*^40^. We also found that selecting this value of *τ*_1_ helped eliminate spurious tracks emergent from the harmonics of the omnipresent 60 Hz interference from power lines. For each signature, the fundamental frequency as well as amplitude and phases of the first two harmonics were recorded.

#### Step 1(b): Cluster into candidates at each time window

At each time window, several harmonic signatures may be identified at each electrode in the previous step. This step of the algorithm clusters the signatures by their fundamental frequency and forms “candidates”; each candidate comprises signatures with the same fundamental frequency, occurring at the same time instant at more than one electrode. Spurious signatures, e.g. due to local noise at an electrode, can be eliminated by this voting method. For tracking *Eigenmannia*, we assume that each candidate corresponds to the measured signal from an individual fish at multiple electrodes at the same time. Since the clustering of candidates is by fundamental frequency, the assumption is that no two fish maintain the same EOD frequency as one another for an extended period of time. Electrosocial behaviors such as Jamming Avoidance Response^41^ and Social Envelope Response^42^ generally serve to isolate each individual’s EOD frequency from conspecifics, except for brief periods of social interaction.

#### Step 1(c): Associate candidate across time

**Figure 7:**
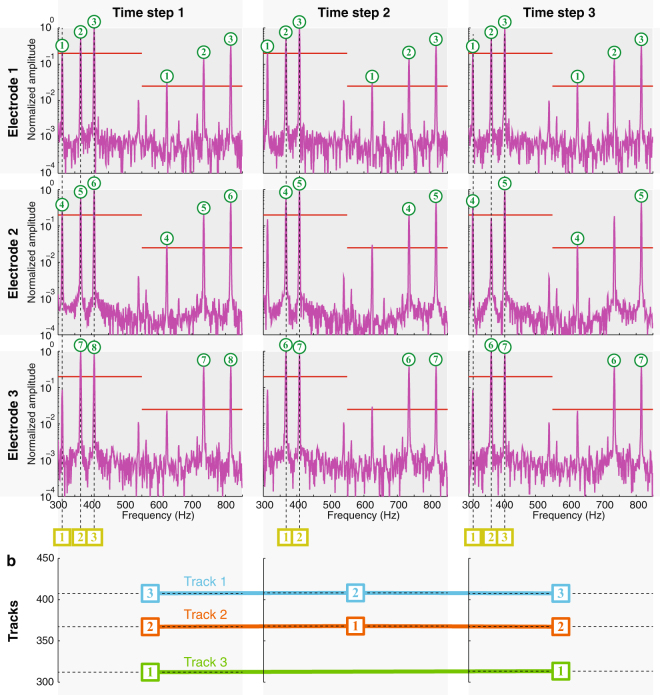
Visual representation of frequency localization algorithm. (**a**) The columns represent three contiguous time windows and the rows represent data measured at three electrodes. Each axis shows normalized (0–1) STFT magnitude vs. frequency (magenta traces). For simplicity, the algorithm is explained using three electrodes. At each time window, for each electrode, the signals whose fundamentals and first harmonics exceed the respective thresholds (red lines) are designated signatures (green circled numbers). At each time window, frequencies at which more than a certain number (in this example, 2) signatures are present, are grouped into candidates (yellow squared numbers). (**b**) Candidates are matched across time windows using the frequency and amplitudes of their fundamental to form tracks (colored lines). These steps are carried out across all time windows, and tracks are created or pruned using confidence criteria.

In the third and final step, candidates are associated across time to form “tracks” (Fig. 7). This association is made using the Hungarian algorithm, a combinatorial optimization algorithm that pairs measurements according to proximity under a distance metric^43^. For our implementation, we used as our metric the Euclidean distance in the combined space of the candidates’ fundamental and second harmonic amplitudes. Candidates in neighboring time instances are associated first. Because of possible frequency crossing and temporal gaps caused during spectral analysis, any remaining candidates two time intervals apart are associated next, and so on, up to a maximum gap of 5 s and a maximum difference in fundamental frequencies of 1 Hz. These thresholds in time and frequency were selected heuristically. The final tracks consist of continuous segments of frequencies, amplitudes, and phases of each putative fish.

We implemented the frequency tracking algorithm in MATLAB, with a GUI that displays the spectrogram from each electrode and the autonomously tracked frequency trajectories. The GUI allows manual edits to the frequency tracks: the user can split, join, or delete tracks. This, along with the ability to set the threshold *τ*_1_ and the range of signature frequencies, provides user input and customization in frequency tracking, and makes sure that the tracked frequencies correspond to the patterns that humans can detect readily when visualizing the spectrogram amplitudes; see Fig. 6.

### Step 2: Spatial localization of a moving dipole source

#### Observations from grid electrodes

The frequency localization algorithm outputs tracks, each of which represents the signal trajectory of a single dipole source. This allows each dipole to be spatially localized independent of other sources. In other words, we spatially track each fish separately, allowing us to omit the superscript *j* (putative fish number) in the derivations that follow.

**Figure 8:**
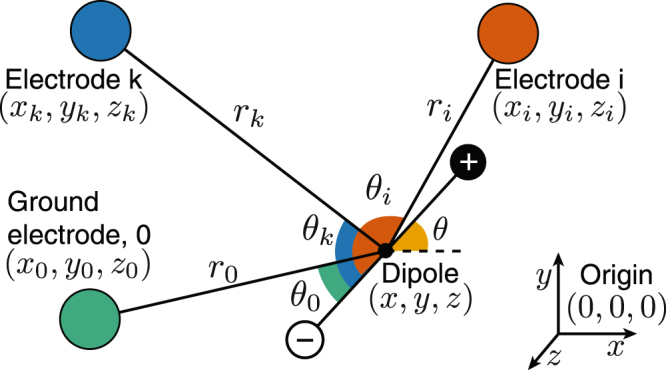
Dipole with two electrodes and ground. The dipole consists of two equal and opposite charges along an axis. The variables used in Equations (6) and (7) are illustrated here. *r*_*i*_, *r*_*k*_ and *r*_0_ are the distances from the dipole axis midpoint, and *θ*_*i*_, *θ*_*k*_ and *θ*_0_ are the angles from the dipole axis, to electrodes *a*, *b* and *g* respectively.

Figure 8 shows a dipole and three electrodes: *i* and *k* are grid electrodes, and 0 is the ground electrode with respect to which all electric potential measurements are made. The vector from the dipole center and each electrodes have lengths *r*_*i*_, *r*_*k*_ and *r*_0_ respectively, and the angles of these vectors from the dipole axis are *θ*_*i*_, *θ*_*k*_ and *θ*_0_ respectively. Note that the dipole and electrodes need not be coplanar; however, as stated earlier, the dipole and the electrode plane is assumed to be parallel.

We obtain amplitudes, phases, and frequencies of the fundamental and second harmonic at each time window from the frequency localization algorithm. As per Equation (3), the amplitude is a function of the polar position and orientation $(r(t),\theta (t))$ of the fish and other fish specific constants. The fundamental phase at an individual electrode by itself does not contain any spatial information as it can vary depending on the phase of the electric field oscillation at the start of the time window. However, if an electrode closer to the positive pole of the dipole has a phase value *ψ*, all other electrodes which are closer to the positive pole will have the same phase. The electrodes which are closer to the negative pole will have the opposite phase, i.e.*ψ* ± *π*. This binary grouping of the electrodes results from the fact that the dipole midline splits the electrode plane into two. Thus, knowledge about this grouping yields information about the orientation of the dipole. In order to leverage this binary (signed) phase information, we define the signed potential amplitude at electrode *i* as:

5 $${\lambda }_{i}={a}_{i}\,{\rm{sign}}(\cos \,{\psi }_{i})$$

Using the grid, we can only measure the potential difference with respect to the ground electrode:

6 $${\lambda }_{i\mathrm{,0}}={\lambda }_{i}-{\lambda }_{0}={a}_{i}\,{\rm{sign}}(\cos \,{\psi }_{i})-{a}_{0}{\rm{sign}}(\cos \,{\psi }_{0})$$

The difference between the voltages at two electrodes *i* and *k* eliminates the effect of the unknown but constant location of the ground electrode:

7 $${\lambda }_{i,k}={\lambda }_{i\mathrm{,0}}-{\lambda }_{k\mathrm{,0}}={a}_{i}\,{\rm{sign}}(\cos \,{\psi }_{i})-{a}_{k}{\rm{sign}}(\cos \,{\psi }_{k})$$

For *n* such electrodes, the voltage measured from the *n*^*th*^ electrode is subtracted from the other (*n* − 1) electrodes and the resulting (*n* − 1) vector is normalized to eliminate fish-specific constants:

8 $${{\rm{\Lambda }}}_{{\rm{ideal}}}(t)=\frac{{({\lambda }_{\mathrm{1,}n}\ldots {\lambda }_{n-\mathrm{1,}n})}^{T}}{||({\lambda }_{\mathrm{1,}n}\ldots {\lambda }_{n-\mathrm{1,}n})||}=h(X(t))$$

This *ideal observation vector* does not depend on the fish-specific constants *K*, *A* or *γ*, but is a function $h(\cdot )$ of the unknown *state X*(*t*) of the dipole (see Fig. 8). This state vector comprises the position of the dipole source with respect to a fixed origin, and angle *θ*(*t*) of the dipole in the horizontal (*x*−*y*) plane:

9 $$X(t)={(x(t),y(t),z(t),\theta (t))}^{T}\mathrm{.}$$

We assume that the actual observations are contaminated with independent, zero-mean observation noise, *v*_obs_:

10 $${\rm{\Lambda }}(t)={{\rm{\Lambda }}}_{{\rm{i}}{\rm{d}}{\rm{e}}{\rm{a}}{\rm{l}}}(t)+{\nu }_{{\rm{o}}{\rm{b}}{\rm{s}}}=h(X(t))+{\nu }_{{\rm{o}}{\rm{b}}{\rm{s}}}$$

If ${f}_{{\rm{o}}{\rm{b}}{\rm{s}}}(\cdot )$ is the probability density of *v*_*obs*_, this also means that the likelihood of a particular observation given the state of the fish is

11 $$p({\rm{\Lambda }}(t)|X(t))={f}_{{\rm{obs}}}({\rm{\Lambda }}(t)-h(X(t\mathrm{)).}$$

Our goal is to find an estimate, $\hat{X}(t)$, for the 4-dimensional state of each dipole, given the (*n* − 1)-dimensional measurement vector ${\rm{\Lambda }}(t)$. Since the relationship between the state and the measurements is through a transcendental equation, this is a highly nonlinear problem, which typically has no closed-form inverse. Even if such an inverse exists, it is still necessary to combine redundant noisy data. To do so, we resort to a particle filter, a statistical estimation technique analogous to other (approximate) Bayesian schemes such as extended or unscented Kalman filters. We chose to use the particle filter because of its ability to handle non-unimodal data.

#### Particle Filter Approach

A particle filter is a statistical estimation technique that can be applied to nonlinear problems with multimodal noise distributions. The state vector of the fish *X*(*t*) evolves in time. We make observations ${\rm{\Lambda }}(t)$ of the system, as described in the previous section. The particle filter uses a set of *N* particles ${\{{X}_{k}(t)\}}_{k\mathrm{=1}}^{N}$, each of which has the same state variables as *X*(*t*), in addition to a weight ${w}_{k}(t)$. Our current knowledge of *X*(*t*) is the posterior distribution given all previous observations, $p(X(t)|{\rm{\Lambda }}\mathrm{(1}:t))$. The assumption is that, given large enough *N*, the set of particles and their respective weights approximates the posterior distribution. That is, each particle is a sample from this posterior distribution and its weight is the probability of drawing that sample.

At time *t*, we compute observation vectors for each of the particles:

12 $${{\rm{\Lambda }}}_{k}(t)=h({X}_{k}(t\mathrm{)).}$$

We also compute the actual observation vector Λ(*t*) from the grid electrode data. From Equation (11), the likelihood of observing the vector Λ(*t*) given the current state of the particle *X*_*k*_(*t*) is computed as:

13 $$p({\rm{\Lambda }}(t)|{X}_{k}(t))={f}_{obs}({\rm{\Lambda }}(t)-h({X}_{k}(t\mathrm{))).}$$

We assume *f*_obs_ to be a zero-mean normal whose covariance matrix is ${\sigma }_{{\rm{obs}}}^{2}$ times an $(n-\mathrm{1)}\times (n-\mathrm{1)}$ identity matrix. The variance ${\sigma }_{{\rm{obs}}}^{2}$ was selected heuristically to give good tracking performance in preliminary lab data.

This likelihood is used to update and re-normalize the weights of the particles from the previous time step:

14 $$\begin{array}{cc}{w}_{k}(t) & ={w}_{k}(t-1)p({\rm{\Lambda }}(t)|{X}_{k}(t)),\\ {w}_{k}(t) & =\frac{{w}_{k}(t)}{\sum _{k=1}^{N}{w}_{k}(t)}.\end{array}$$

The state estimate at time *t*, $\hat{X}(t)$, is the weighted mean of the particles:

15 $$\hat{X}(t)=\sum _{k\mathrm{=1}}^{N}{w}_{k}(t){X}_{k}(t\mathrm{).}$$

The particles at time *t* evolve via a simple random motion model:

16 $${X}_{k}(t+\mathrm{1)}={X}_{k}(t)+{\nu }_{mot},$$

where *v*_*mot*_ is drawn from a 4-dimensional normal distribution with zero mean and variances which were tuned heuristically to ensure good tracking performance in laboratory data. We found that the results were not sensitive to this value, and we were able to change *v*_*mot*_ by an order of magnitude without substantive change to tracking performance.

#### Specifics of filter implementation

We implemented the particle filter algorithm as described in Arulampalam *et al*.^30^, with the parameters and modifications as described below.

We used *N* = 2.5 × 10^5^ particles. At the first time step, the initial states of the particles were sampled from a non-informative (uniform) prior distribution in the state space. The weights of the particles, *w*_*k*_, are set to $1/N$ at the first time step. To achieve reasonable particle density, we constrained the state space. For *x* and *y*, the limits are the tank boundaries in lab data, and twice the dimensions of the grid for field data. The *z* state can take values between 0–3 meters from the plane of the grid of electrodes for both the lab data and field data. Note that this restriction in state is relaxed for subsequent time steps: particles are permitted to cross the constraint threshold if that is what the motion model dictates.

One common problem that arises during particle filter implementation is sample impoverishment, where most particles end up having (near) zero weights after a few iterations. In order to counter this, we compute the effective number of particles^30^:

17 $${N}_{{\rm{eff}}}=\frac{1}{\sum _{k\mathrm{=1}}^{N}{w}_{k}{(t)}^{2}}$$

When *N*_eff_ falls below a threshold, which we set to $N/2$, we trigger a resampling operation. 50% of the particles are resampled from the existing distribution, as represented by the particles and their weights. 45% of the particles are “locally sampled”, i.e. they are normally distributed around the current estimate, $\hat{X}(t)$. 5% of the particles are “globally sampled” from the same non-informative uniform prior distribution in the state space used at the first time step.

During tracking, the particles often converge to a small region of state space around the fish as desired, and consequently, the density of particles becomes sparse in most of the state space. *Eigenmannia* sometimes perform rapid “darting” movements, swimming to high velocities with high acceleration for a short period of time. Such movements are not readily captured by the motion model which is tuned to capture smooth, not ballistic, motion. If the fish moves rapidly in this way into a region where there are few particles, the distribution of particles will not keep pace, and the estimate will diverge. The obvious way to counter this would be to obtain spatial estimates at a temporal rate fast enough to capture these darting movements. However, the hardware EOD sampling rate, combined with the length of the time window needed to encompass sufficient number of cycles of the EOD for obtaining a discernable spectrum, limits the temporal rate of frequency and spatial tracking. Instead, we deploy the locally and globally sampled particles to maintain a sufficient particle density in the state space to capture these quick movements.

### Laboratory experiments

In order to validate our tracking algorithm, we performed experiments of fish swimming in a large laboratory tank. We used a 3 × 3 electrode grid with an inter-electrode spacing of 30 cm in an acrylic tank of dimensions 1.5 m × 1.2 m × 0.3 m (Fig. 1). The tank was filled to a depth of 28 cm with water, whose conductivity was maintained between 100–250 *μ* S/cm. The PVC support of the grid was at the water surface, and the electrodes were lowered to a depth of ~10 cm below the surface. Fish were released into the tank and acclimatized for several hours prior to recording data.

A camera (Logitech C920) with a ring illuminator captured an overhead view of the tank at 30 frames per second. Retroreflective markers were placed on the top surfaces of the electrodes as well as the four corners of the tank. These markers were isolated and identified post-hoc through image processing to detect the position and orientation of the tank and electrodes in the camera frame. Electric potential measurements from the electrodes were digitized at 20 kHz and recorded using a Spike2 data acquisition device (CED, Cambridge, U.K.). The acquisition device also triggered a blinking LED in the field of view of the camera, the timing of which was used to synchronize between the electrode recordings and camera frames. We performed two types of laboratory experiments: TUBE and FREE.

In the TUBE condition, each fish was confined to a tube of length 20 cm with plastic mesh sides and ends. The tubes were placed horizontally at a depth of 10–15 cm at different locations and orientations relative to the electrode grid. Two retroreflective markers were placed on either end of each tube, which enabled us to track the position and orientation of the tubes in the camera frame. We performed 15 trials with a single fish in a tube, with the tube at different positions and orientations across the tank both within the grid perimeter and outside the grid. We also performed 40 trials with three fish in three separate tubes (e.g. Fig. 2(a)), arranged from being immediately adjacent to each other to being across the tank from each other, across a variety of relative orientations. The position and orientation of the tubes tracked from a single frame of the overhead video is considered the *video-tracked pose*, and the mean of the estimates from our algorithm is considered the *electrode-tracked pose*. (See supplementary figures for more examples. All TUBE data are made available as part of this publication).

In the FREE condition, fish were released into the tank and allowed to swim freely. When the lights were on, fish preferred to remain at the edge of the tank, outside the grid. To maintain movement, the experimenter used a clear acrylic rod to induce the fish to swim. For our analysis, we chose epochs where the fish were moving for a majority of the epoch. We performed trials with a single fish in the tank (e.g. Fig. 2(b)), and with groups of three fishes. The head and tail of each fish were manually clicked every 10^*th*^ video frame. The clicked points are used to compute the position and orientation of the fish, and form the *video-tracked poses*. The estimates from our algorithm at the same time instances as the clicked video frames are the *electrode-tracked poses*. (See supplementary videos for more examples. All FREE data are made available as part of this publication).

### Statistical Methods

We used a shuffling (Monte-Carlo permutation) procedure in order to analyze the performance of our tracking algorithm. For the TUBE and FREE trial types, we compiled all pairs of poses (X-Y positions and orientations) of all fish tracked through overhead video and our electrode-based tracking method. Each pose pair consisted of one video-tracked pose of one fish for one frame of the overhead video, and its corresponding electrode-tracked pose. There were 135 pose pairs for the TUBE dataset (using a single video frame per trial for each fish) and 3222 pose pairs for the FREE dataset (using every tenth video frame from each trial for each fish). In each shuffling iteration, the video-tracked poses were randomly permuted, and position and orientation errors to their corresponding electrode-tracked poses were computed. These iterations were repeated 100,000 times. The set of cumulative errors from the iterations form the *shuffled cumulative error distribution*. The set of root-mean-square (RMS) errors from each iteration form the *shuffled RMS error distribution*. The data were also divided into instances where the video-tracked positions are within the grid (TUBE: 73, FREE: 2081) or outside the grid (TUBE: 62, FREE: 1141). The shuffling procedure was also repeated for these subsets, creating three cumulative error and three RMS error distributions.

We compared the true (non-shuffled) cumulative error to the shuffled cumulative error distribution (Fig. 2c,d). We also compared the true (non-shuffled) RMS error to the shuffled RMS error distribution (Fig. 3). This was to test the null hypothesis that the tracking error of the actual data was statistically no different from a random permutation of the locations of the fish.

### Data availability

The laboratory and field data associated with this publication, as well as an archived version of the code used to process these datasets are made available through the Johns Hopkins University Data Archive. doi:10.7281/T1/XTSKOW.

### Code availability

The code for the tracking algorithm is available in the public git repository: https://github.com/manusmad/fishtracker.

## Electronic supplementary material

Video - Single free fish tracking, example 1

Video - Single free fish tracking, example 2

Video - Single free fish tracking, example 3

Video - Three free fish tracking, example 1

Video - Three free fish tracking, example 2

Video - Three free fish tracking, example 3

Video - Three free fish tracking, example 4

Supplementary figures and video descriptions
