# Supplementary material for: High-resolution behavioral mapping of electric fishes in Amazonian habitats
Source: Sci Rep. 2018 Apr 11;8:5830. doi: 10.1038/s41598-018-24035-5 (PMC5895713; doi:10.1038/s41598-018-24035-5)
Supplement: Supplementary file 8 — Supplementary figures and video descriptions [file 41598_2018_24035_MOESM8_ESM.pdf]

# High-resolution behavioral mapping of electric fishes in Amazonian habitats

Manu S. Madhav[1,*,†], Ravikrishnan P. Jayakumar[2,†], Alican Demir[2], Sarah A. Stamper[2], Eric S. Fortune[3] & Noah J. Cowan[2]

† Authors contributed equally to the work.
**1** Mind/Brain Institute, Johns Hopkins University, Baltimore, Maryland, U.S.A.
**2** Mechanical Engineering Department, Johns Hopkins University, Baltimore, Maryland, U.S.A.
**3** Department of Biological Sciences, New Jersey Institute of Technology, Newark, New Jersey, U.S.A.

## Supplementary figures

### Field trials

Figs. 1,2,3: A grid of 8 electrodes deployed in at a field site in Brazil (right) recorded signals from three fish restricted in tubes, while other free electric fish swam within the grid. The spectrogram of the recorded data (left) reveals several tracks, including those belonging to the restrained fish (marked as 1-3). The mean and two standard deviations of the position estimate of the restrained fish is marked using circles, and the mean and two standard deviations of the orientation estimate is marked using wedges within the circles.

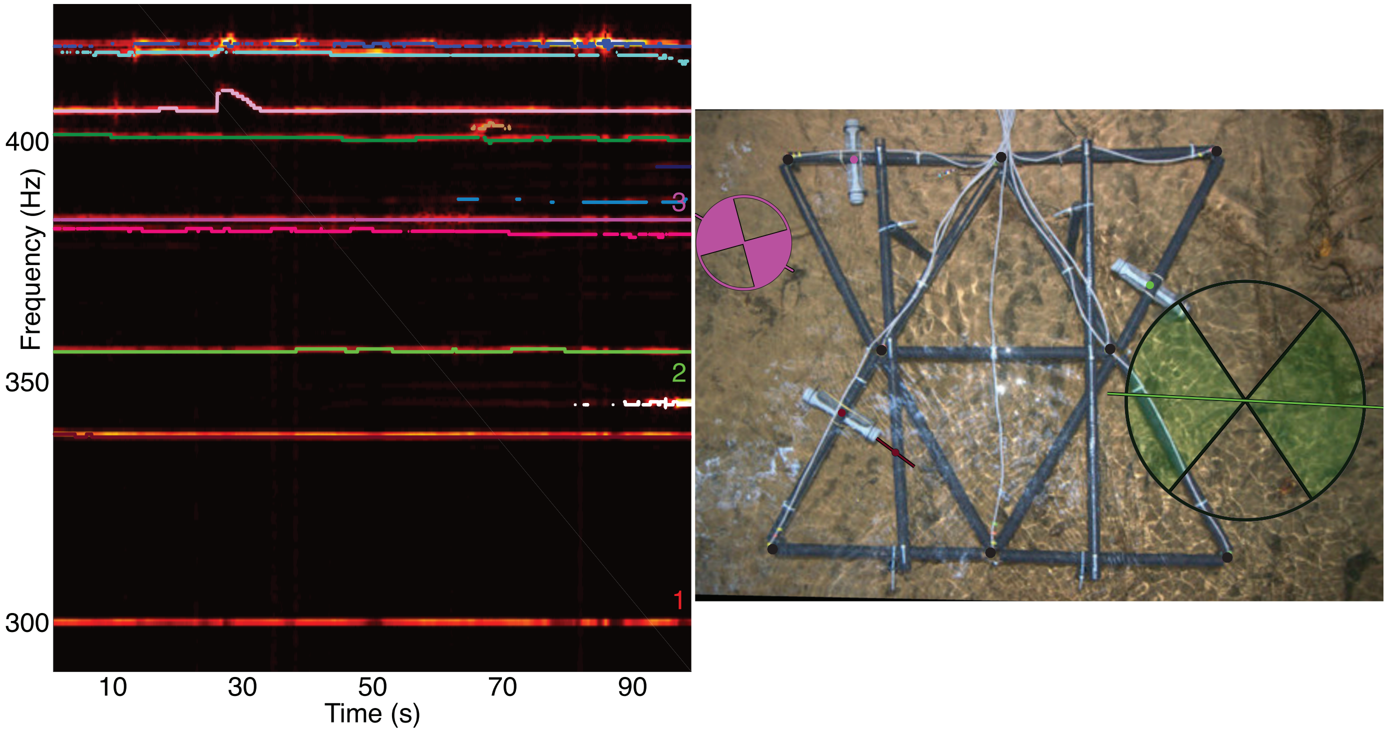


Figure 1: Field trial #2

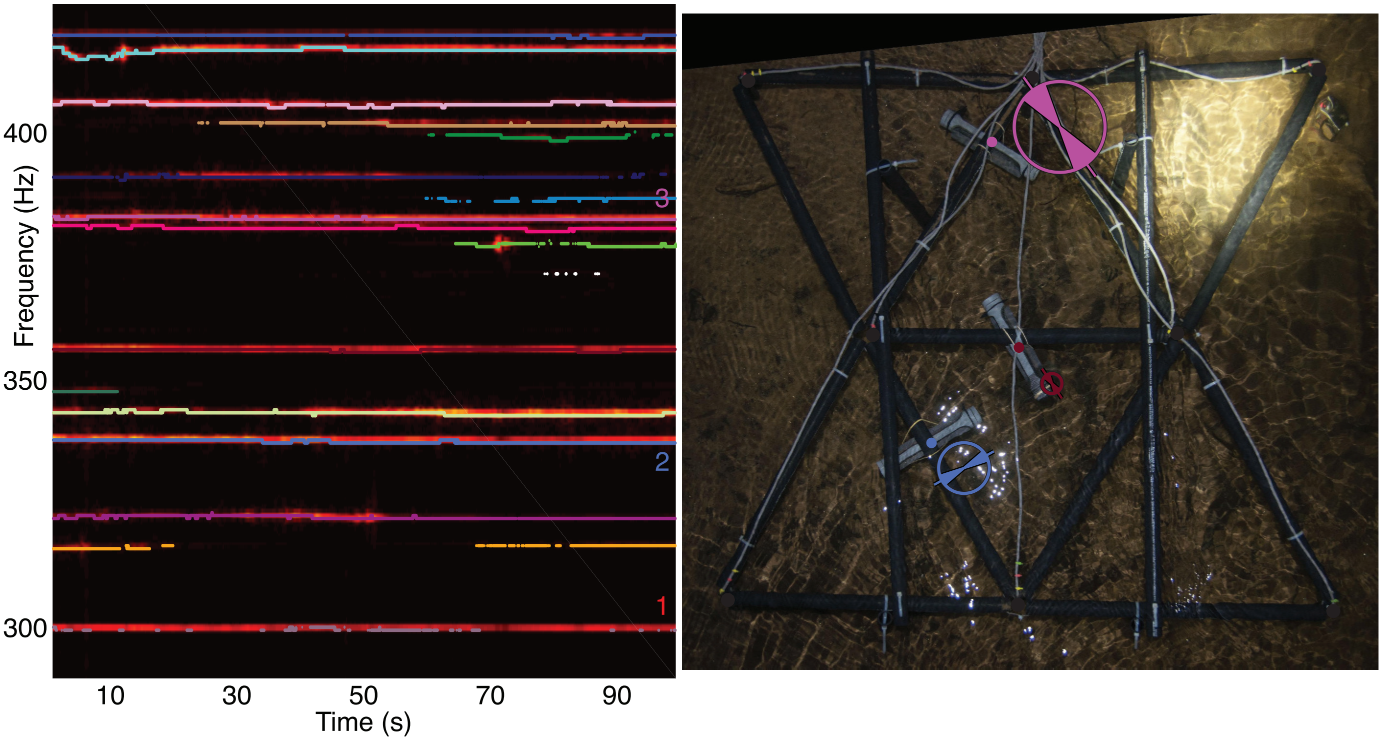


Figure 2: Field trial #3

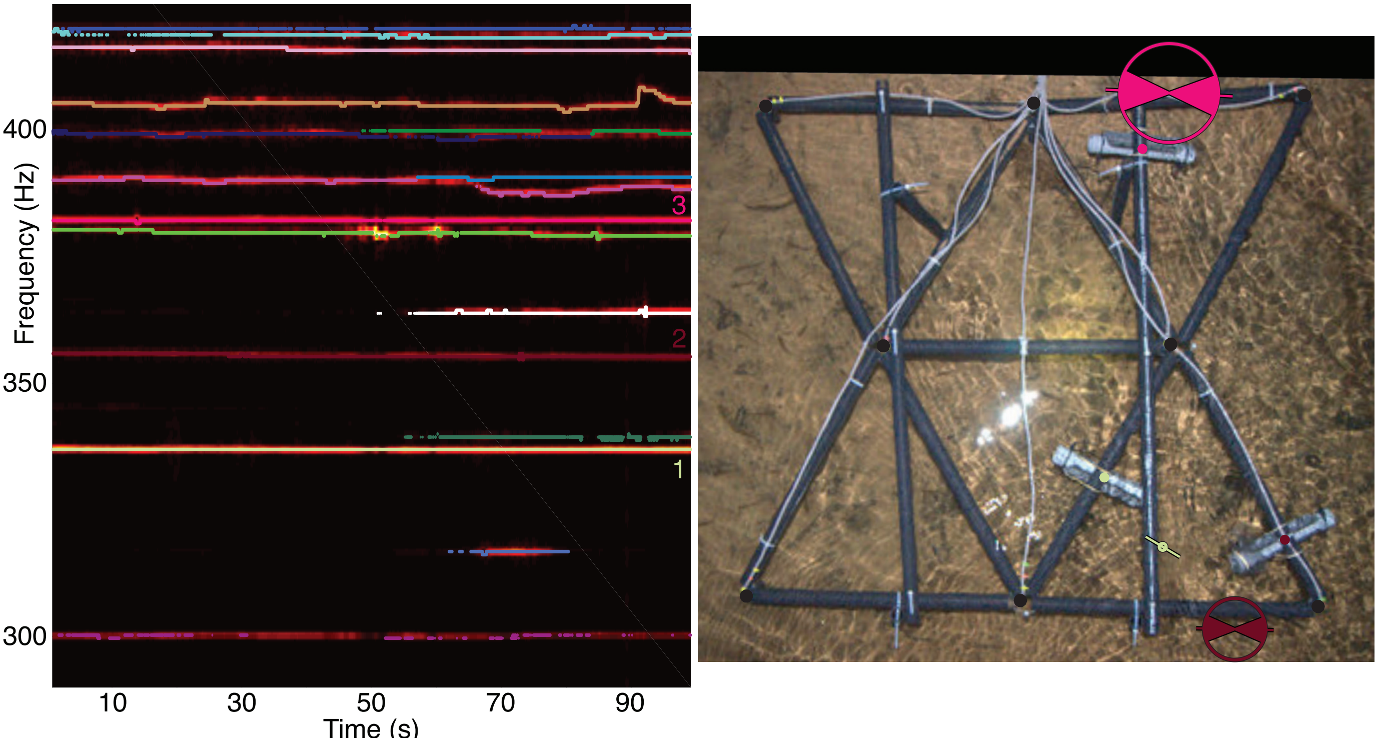


Figure 3: Field trial #4

## Laboratory trials: TUBE, single fish

Figs. 4,5,6: Overhead view of a grid of 9 electrodes in the laboratory tank (right), which recorded signals from one fish restricted in a tube. The spectrogram of the recorded data (left) reveals the frequency track of the fish. The mean and two standard deviations of the position estimate of the restrained fish is marked in the overhead view using a circle. The mean and two standard deviations of the orientation estimate is marked using a wedge within the circle.

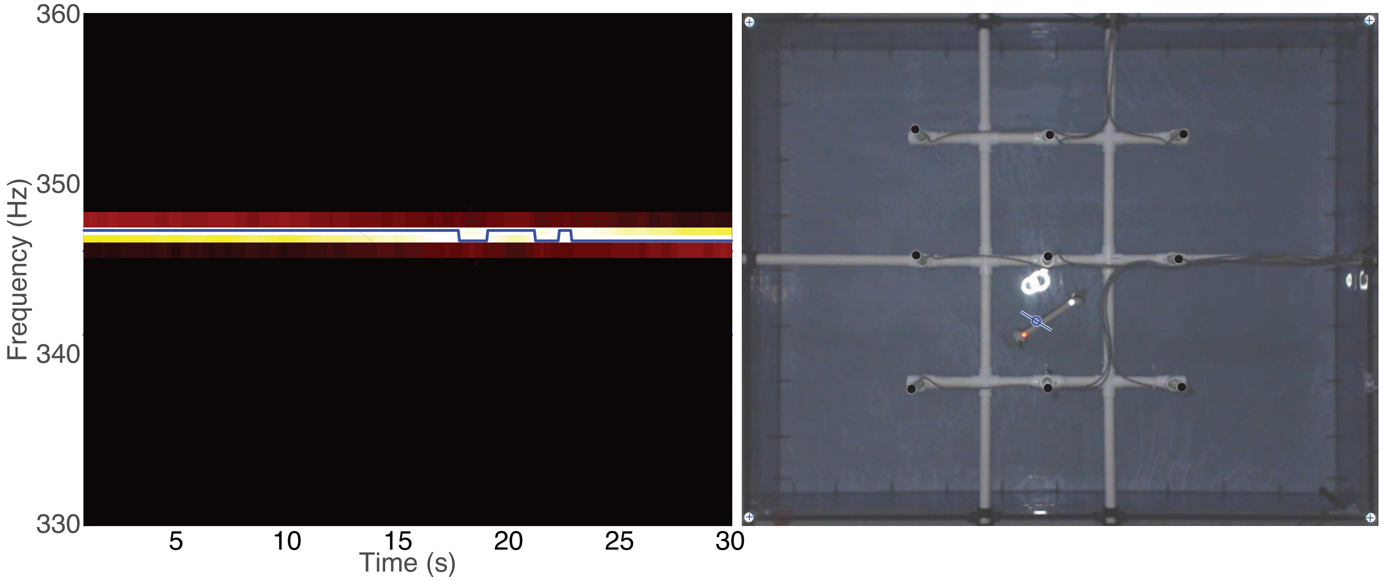


Figure 4: TUBE trial, Single fish, Trial #3

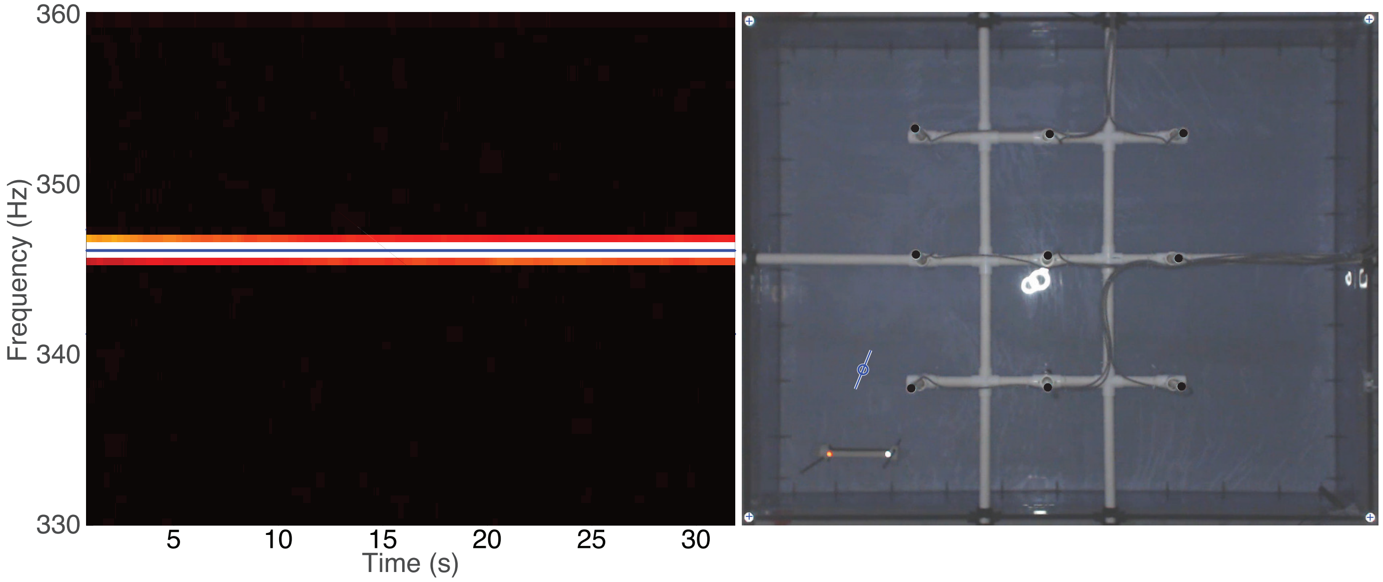


Figure 5: TUBE trial, Single fish, Trial #4

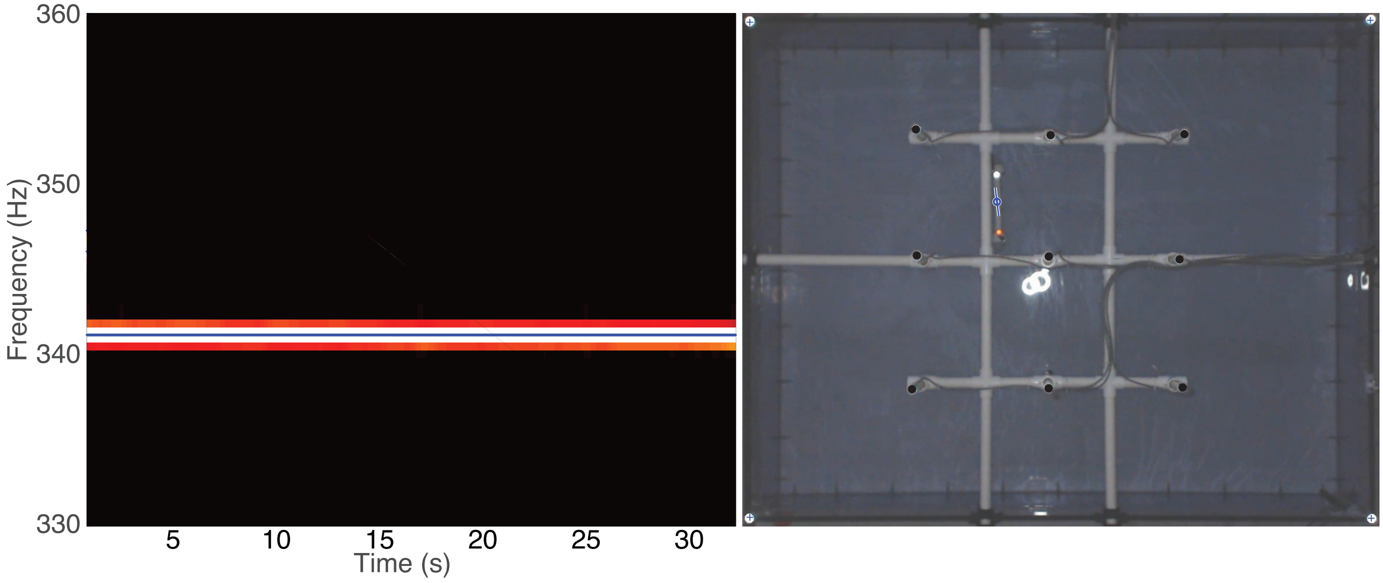


Figure 6: TUBE trial, Single fish, Trial #11

## Laboratory trials: TUBE, three fish

Figs. 7,8,9: Overhead view of a grid of 9 electrodes in the laboratory tank (right), which recorded signals from three fish restricted in tubes. The spectrogram of the recorded data (left) reveals frequency tracks of the fish. The mean and standard deviation of the position estimates of the restrained fish are marked in the overhead view using circles. The mean and standard deviation of the orientation estimates are marked using wedges within each circle.

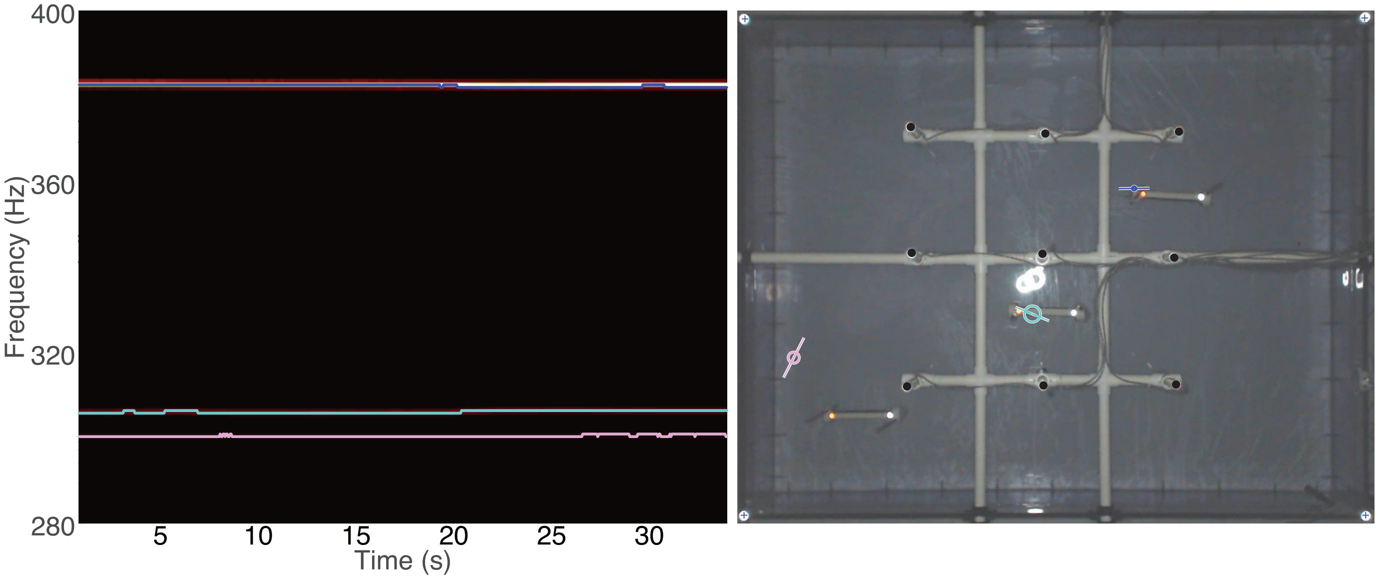


Figure 7: TUBE trial, Three fish, Trial #1

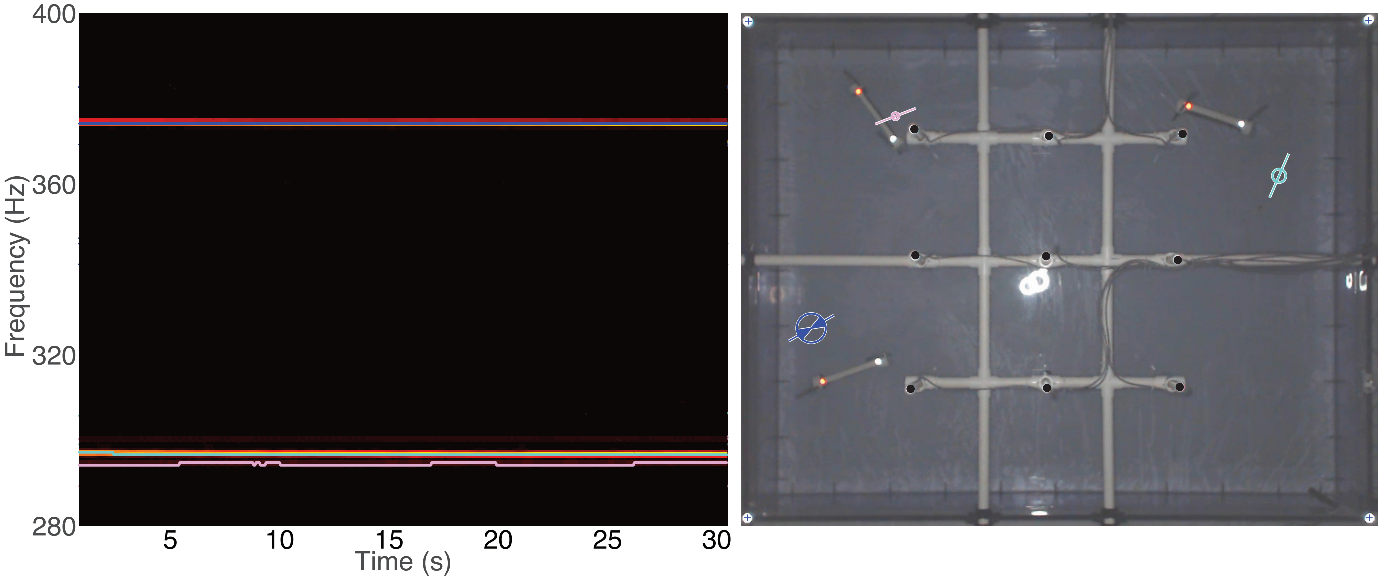


Figure 8: TUBE trial, Three fish, Trial #25

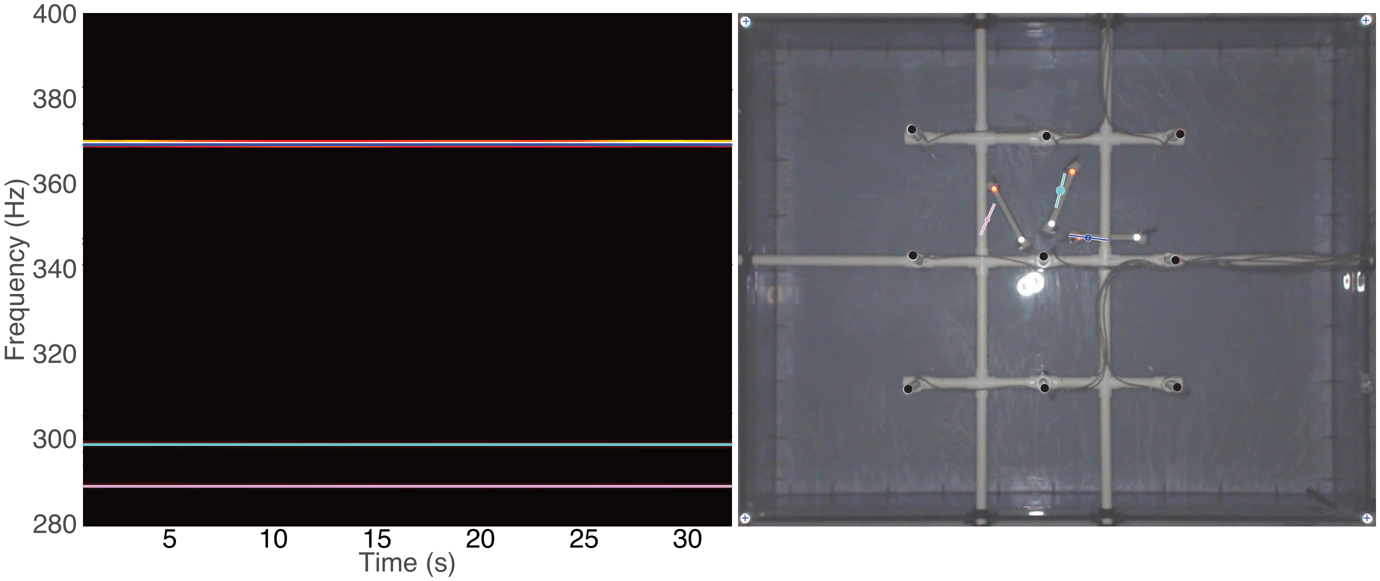


Figure 9: TUBE trial, Three fish, Trial #38

## Supplementary videos: FREE, single fish

There are three example video files showing frequency and spatial tracking of a single freely swimming weakly electric fish:
`140406_002_01m07s_01m27s_video.mp4`
`140406_002_03m34s_03m54s_video.mp4`
`140406_002_07m05s_07m55s_video.mp4`

In each video, the left panel shows the mean spectrogram from all electrodes, with the tracked frequency trace overlaid in blue. The right panel shows the overhead view of a grid of 9 electrodes in the laboratory tank, which recorded signals from one fish that can be seen swimming within the grid. The instantaneous position and orientation estimate of the fish as tracked by our algorithm is shown by the blue ellipse, whose position denotes the position estimate, and whose major axis denotes the orientation estimate.

## Supplementary videos: FREE, three fish

There are four example video files showing frequency and spatial tracking of three freely swimming weakly electric fish:
`140422_001_05m50s_06m09s_video.mp4`
`140422_001_08m40s_09m05s_video.mp4`
`140422_001_09m12s_09m37s_video.mp4`
`140422_001_09m50s_11m00s_video.mp4`

In each video, the left panel shows the mean spectrogram from all electrodes, with the tracked frequency traces overlaid in blue, cyan, and pink. The right panel shows the overhead view of a grid of 9 electrodes in the laboratory tank, which recorded signals from three fish that can be seen swimming within the grid. The instantaneous position and orientation estimate of the fish as tracked by our algorithm are shown by the correspondingly colored ellipses, whose positions denote the position estimates, and whose major axes denote the orientation estimates.
